# Hypothermia therapy for the treatment of acute myocardial infarction: A protocol for systematic review and meta-analysis

**DOI:** 10.1097/MD.0000000000027338

**Published:** 2021-09-24

**Authors:** Fen Jiang, Defei Zeng, Kongyu Xing, Xiaoli Yang

**Affiliations:** a International School of Nursing, Hainan Medical University, Hainan, China; b Department of Cardiology, The First Affiliated Hospital of Hainan Medical University, Hainan, China.

**Keywords:** complications, hypothermia, meta-analysis, myocardial infarction

## Abstract

**Background:**

In patients with acute myocardial infarction (AMI) receiving percutaneous coronary intervention (PCI), the role of systemic therapeutic hypothermia remains controversial. We performed a protocol for systematic review and meta-analysis to investigate the effect of systemic therapeutic hypothermia in patients with AMI receiving PCI.

**Methods:**

This study will use the Cochrane Library, Web of Science, PubMed, Embase, Allied and Complementary Medicine Database, China Biomedical Literature Database, China National Knowledge Infrastructure, China Science and Technology Journal Database, Wanfang Database, and Ongoing Clinical Trials Database. The search terms were hypothermia, cooling, myocardial infarction, myocardial ischemia and acute coronary syndrome. Quality assessment of the included studies was evaluated using the Cochrane risk of bias assessment tool. Statistical analyses were performed using RevMan 5.4 software.

**Results:**

The findings of this study will be submitted to peer-reviewed journals for publication.

**Conclusion:**

This systematic review will provide evidence to determine whether hypothermia therapy is an effective and safe intervention for patients with AMI receiving PCI.

Registration number: 10.17605/OSF.IO/9XJSB.

## 1 Introduction

Cardiovascular disease poses tremendous burden on public health, as well as the global economy.^[1,2]^ According to the World Health Organization, an estimated 17.5 million people died from cardiovascular disease in 2012, accounting for 31% mortality globally with an annual cost of $193.1 billion in healthcare management and about $123 billion in productivity loss as a result of premature death.^[3,4]^ An acute myocardial infarction (AMI) is a leading cause of morbidity and mortality worldwide with known complications including congestive heart failure, functional and structural myocardial abnormalities, reinfarction, and death.^[5–7]^ Percutaneous coronary intervention (PCI) represents the therapy of choice for reperfusion of patients with ST-segment elevation AMI.^[8,9]^ However, even after timely restoration of blood flow at epicardial level, a substantial proportion of AMI patients develop extensive myocardial necrosis.^[10]^ The ischemic process represents one of the most important determinants of myocardial damage in the infarct-related area, being modulated by means of several protective modalities including pharmacological agents, pre- or post-conditioning, and hypothermia.^[11]^

The rationale for hypothermia in patients with AMI lies on the reduction of energy consumption at cardiac level, which, in turn, has been consistently associated with reduced myocardial infarct size in several animal studies.^[12]^ However, a number of randomized trials, comparing different strategies for systemic therapeutic hypothermia adjunctive to PCI versus standard of care in patients with AMI, failed to translate these experimental findings in consistent beneficial effects in humans; indeed, while some trials demonstrated the clinical benefit of hypothermia at least in certain subgroups of patients, other trials suggested harm. In this regard, the role of systemic therapeutic hypothermia in this setting remains to be defined.

To gain more insight into this topic, we performed a protocol for systematic review and meta-analysis to investigate the effect of systemic therapeutic hypothermia in patients with AMI receiving PCI.

## 2 Methods

### 2.1 Study registration

The protocol of this review was registered in OSF (OSF registration number: 10.17605/OSF.IO/9XJSB). It is reported to follow the statement guidelines of preferred reporting items for systematic reviews and meta-analyses protocol.^[13]^ Since this study is on the basis of published studies, ethical approval is not required.

### 2.2 Searching strategy

This study will use the Cochrane Library, Web of Science, PubMed, Embase, Allied and Complementary Medicine Database (AMED), China Biomedical Literature Database (CBM), China National Knowledge Infrastructure (CNKI), China Science and Technology Journal Database (VIP), Wanfang Database, and Ongoing Clinical Trials Database. There is no definite time limit for the retrieval literature, and the languages are limited to Chinese and English. We will consider articles published between database initiation and September 2021. The search terms were hypothermia, cooling, myocardial infarction, myocardial ischemia and acute coronary syndrome.

### 2.3 Eligibility criteria

1. Participants: patients who were clearly diagnosed with AMI, and gender and age were not limited.
2. Study type: randomized controlled trials, not limited to blind, language limited to Chinese and English.
3. Interventions: the experimental group was treated with systemic therapeutic hypothermia combined PCI and control group was treated with standard care combined PCI.
4. Outcomes: the primary outcome of the current study was all-cause death. The secondary outcome was infarct size as measured by cardiac imaging. Other outcomes were recurrent MI, ischemia-driven target vessel revascularization and major adverse cardiovascular events.
5. Exclusion criteria:
  (a) For repeated publications, select the literature with the most complete data.
  (b) The type of publications were comments, experience presentations, conference articles, reviews, or case reports.

### 2.4 Data selection

First, two investigators used Endnote X9 software to conduct a preliminary assessment of the title and abstract of each document in the database based on the established criteria for inclusion in the study to select eligible studies. After a preliminary assessment, the full text of the selected literature was evaluated, and the uncontrolled study, no randomization, inconsistent evaluation criteria, and similar data were excluded. Finally, the final included literature was exchanged and checked by researchers. If the two researchers disagree on the results of a study or eventual inclusion, we will resolve it through discussion or consultation with a third person.

### 2.5 Data extraction

Before data collection, the study team built a data extraction sheet. Two authors separately collected relevant information from each eligible study. The data extraction table mainly includes the following contents: research title, first author, year of publication, sample size, duration of disease, intervention measures, outcome indicators, adverse reactions, and so on. If a study has unclear or inadequate information, we will attempt to contact the authors via email.

### 2.6 Risk of bias assessment

Two investigators will separately assess the risk of bias of the included studies using the Cochrane risk of bias assessment tool. The evaluation of each study mainly included the following seven aspects: random sequence generation, allocation hiding, blinding of participants and personnel, blinding of outcome assessment, incomplete outcome data, incomplete outcome data, selective outcome reporting, and other biases. Finally, the bias of the study will be rated on three levels: “low,” “high,” and “ambiguous.” These even domains will be separately appraised by two reviews, and discrepancies will be addressed by consulting a third reviewer.

### 2.7 Data synthesis

In this study, we will apply RevMan 5.4 software for statistical analysis. The risk ratio and 95% confidence intervals (CIs) were collected for enumeration data, while the mean difference or standardized mean difference and 95% CIs were used to calculate continuous outcome data. The heterogeneity of the data was tested by calculating I^2^ statistics. The study was not considered to have a large heterogeneity when the *I*^2^ value was <50%. When the *I*^2^ value exceeded 50%, there was significant statistical heterogeneity among the trials. When there is homogeneity in the merged outcome results across sufficient studies, a meta-analysis will be conducted. Otherwise, we performed a subgroup analysis to explore the causes of the heterogeneity.

## 3 Discussion

Notwithstanding the encouraging preclinical data,^[14,15]^ randomized trials investigating the outcomes of hypothermia in patients with AMI receiving PCI have been largely inconclusive. Indeed, although hypothermia has proven to be feasible and safe in this clinical setting, its clinical benefit has yet to be demonstrated.^[16]^ As shown in animal trials, the precise schedule of hypothermia is of paramount importance, mandating immediate initiation of hypothermia and achieving therapeutic levels prior to revascularisation although the optimal target temperature is still a matter of debate.^[17]^ Most previous studies already used a target temperature of 35.0°C at the time of reperfusion but achieving this has remained a major challenge in all clinical trials. Future trials should specifically examine the possible causative association between hypothermia, platelet aggregation, and subsequent risk of ST in contemporary practice, addressing whether there is a particular hazard with certain temperature management systems as compared to others.

## Author contributions

**Conceptualization**: Defei Zeng.

**Data curation**: Defei Zeng.

**Funding acquisition**: Xiaoli Yang.

**Investigation**: Kongyu Xing.

**Methodology**: Kongyu Xing.

**Writing** – **original draft**: Fen Jiang.

**Writing** – **review & editing**: Fen Jiang.
